# Use of Blue-Green Fluorescence and Thermal Imaging in the Early Detection of Sunflower Infection by the Root Parasitic Weed *Orobanche cumana* Wallr.

**DOI:** 10.3389/fpls.2017.00833

**Published:** 2017-05-18

**Authors:** Carmen M. Ortiz-Bustos, María L. Pérez-Bueno, Matilde Barón, Leire Molinero-Ruiz

**Affiliations:** ^1^Department of Crop Protection, Institute for Sustainable Agriculture, CSICCordoba, Spain; ^2^Estación Experimental del Zaidín, CSICGranada, Spain

**Keywords:** broomrape, carotenoids, early diagnosis, *Helianthus annuus* L., multicolor fluorescence, thermal detection

## Abstract

Although the impact of *Orobanche cumana* Wallr. on sunflower (*Helianthus annuus* L.) becomes evident with emergence of broomrape shoots aboveground, infection occurs early after sowing, the host physiology being altered during underground parasite stages. Genetic resistance is the most effective control method and one of the main goals of sunflower breeding programmes. Blue-green fluorescence (BGF) and thermal imaging allow non-destructive monitoring of plant diseases, since they are sensitive to physiological disorders in plants. We analyzed the BGF emission by leaves of healthy sunflower plantlets, and we implemented BGF and thermal imaging in the detection of the infection by *O. cumana* during underground parasite development. Increases in BGF emission were observed in leaf pairs of healthy sunflowers during their development. Lower BGF was consistently detected in parasitized plants throughout leaf expansion and low pigment concentration was detected at final time, supporting the interpretation of a decrease in secondary metabolites upon infection. Parasite-induced stomatal closure and transpiration reduction were suggested by warmer leaves of inoculated sunflowers throughout the experiment. BGF imaging and thermography could be implemented for fast screening of sunflower breeding material. Both techniques are valuable approaches to assess the processes by which *O. cumana* alters physiology (secondary metabolism and photosynthesis) of sunflower.

## Introduction

Sunflower (*Helianthus annuus* L.) oil is a major commodity in world trade mainly in Europe, where 60% of the total world production is obtained every year (FAOSTAT, 2016). The main biotic constraint for sunflower oil production in all the countries where sunflowers are grown -with the only exception of the Americas- is broomrape, caused by the achlorophyllous parasitic plant *Orobanche cumana* Wallr. Currently, *O. cumana* is present in 15–20% of the area cropped to sunflower in the world, causing close to 100% yield losses under high infestations (Fernández-Martínez et al., 2015). After parasite seed germination in the soil early after sowing, penetration of its intrusive cells into the host root tissues triggers their division, leading to the formation of a subterranean shoot that grows outside the root of sunflower. Then, parasite shoots emerge from the soil and form a flowering spike that rapidly produces a large amount of tiny seeds. *Orobanche cumana* parasitizes sunflower all through the crop growing season, and flowering of both, host and parasite, are coincident in time (Molinero-Ruiz et al., 2015).

Vascular connections established between broomrapes (*Orobanche* spp. and *Phelipanche* spp.) and their hosts force a complete disruption of the vascular system of the latter during the initial weeks of parasitism (Westwood, 2013). As a result, strong sink strength diverts water and nutrients necessary for the growth of the host (Stewart and Press, 1990). From the emergence of *O. cumana* shoots aboveground onwards, a reduced growth of sunflower is evident (Alcántara et al., 2006) and the decrease of the yield is due to absent or smaller sized capitula, low number and small seeds, and even plant death (Molinero-Ruiz et al., 2015). Thus, most of the metabolic imbalance and water flow impairment might be produced irreversibly in sunflower during underground parasite stages. Since it is physiologically closely linked to sunflower, *O. cumana* is extremely difficult to control in its parasitic stage. Some herbicide treatments may be effective, but depend on the development of herbicide resistance in the crop (Molinero-Ruiz et al., 2015). With regard to the free-standing stage of the parasite, thousands of tiny seeds are produced each season, their longevity contributing to the build-up of populations in soils. Since soil fumigation and solarisation are not economically feasible, use of inducers of suicidal broomrape germination requires an appropriate application technology and agronomic practices and biological control agents have limited efficacy, the effective control of *O. cumana* relies on genetic resistance (Molinero-Ruiz et al., 2015; Fernández-Aparicio et al., 2016).

Imaging techniques, especially blue-green fluorescence and thermal imaging among others, are powerful tools for use in plant stress detection (Lichtenthaler and Miehé, 1997; Chaerle and Van Der Straeten, 2000), including determination of plant diseases (Nilsson, 1995). They are based on the assessment of optical properties of plants within different regions of the electromagnetic spectrum and are able to utilize information beyond the visible range (Chaerle and Van Der Straeten, 2000). Under UV-excitation, plants can emit a wide fluorescence spectrum ranging from about 400 to 800 nm. This spectrum is the sum of two distinct types of fluorescence: blue-green fluorescence (BGF) characterized by a peak around 440 nm (F440) and a shoulder near 520 nm (F520), and fluorescence in the red and far-red regions with two characteristic peaks at about 680 nm (F680) and 740 nm (F740), respectively. The intensity of the BGF after UV light excitation is constant on a short time scale (minutes) and it has been proven to be very sensitive to single stress factors in plants (Buschmann and Lichtenthaler, 1998; Buschmann et al., 2000). The BGF from intact leaves is emitted by cinnamic acids, mainly ferulic acid (Morales et al., 1996), covalently bound to the cell walls of the epidermis, and by other phenolic compounds (Buschmann et al., 2000). In addition, BGF can emanate also from other secondary metabolites as reviewed by Cerovic et al. (1999). Moreover, quantitative variation of plant secondary metabolites associated with BGF emission can be the consequence, among others, of leaf aging (Morales et al., 2005), water stress (Kautz et al., 2014) or pathogen infection (Chaerle et al., 2007; Granum et al., 2015; Pérez-Bueno et al., 2015). Additionally, F680 and F740 are emitted by chlorophyll *a* (Chl *a*) and they are dependent on the pigment content and other factors such as optics of the leaf (Buschmann and Lichtenthaler, 1998; Gitelson et al., 1998).

On the other hand, radiation emitted by plants in the thermal infrared range from 8 to 12–14 μm can be detected by thermographic and infrared cameras. Infrared thermography is an indicator of transpiration and stomatal conductance (Eaton and Belden, 1929; Jackson et al., 1977; Nilsson, 1995). It has been correlated, among others, with plant water status (Zarco-Tejada et al., 2012; Raza et al., 2014; Grant et al., 2016; Mangus et al., 2016) and canopy microclimate (Lenthe et al., 2007; Leuzinger and Korner, 2007), as well as with early infections by airborne (Lindenthal et al., 2005; Baranowski et al., 2015; Pérez-Bueno et al., 2015) and soilborne (Wang et al., 2012; Calderón et al., 2013; Granum et al., 2015) plant pathogens.

Both fluorescence and thermal imaging of whole leaves and whole plants have become essential techniques from the perspective of non-destructive monitoring of plant diseases from far (remote sensing) and from near distance, since sensors are sensitive to physiological disorders in plants associated with pathogen attack and with disease resulting from that attack (Buschmann et al., 2000; Chaerle and Van Der Straeten, 2000; Mahlein, 2016). Techniques for near distance monitoring of plant diseases can have a direct application in plant phenotyping of breeding programmes as those of sunflower. In this regard, fluorescence imaging in the red and far-red region has been recently applied in the early diagnosis of the infection of sunflower by *O. cumana* (Ortiz-Bustos et al., 2016a).

The objectives of this study were to: (a) analyse the distribution of BGF emitted by sunflower leaves during early growth stages of healthy sunflower plants in order to identify the adequate areas and times for BGF imaging, (b) evaluate the use of BGF imaging as an indicator of the infection of sunflower by *O. cumana* during initial underground development of the parasite and compare the BGF information with the pigments concentration in leaves, and (c) analyse the effect of underground infection by *O. cumana* in early stages of sunflower growth using thermal imaging of the leaves.

## Materials and methods

### Growth of healthy sunflower

Four sunflower seeds of the inbred line NR5 were surface sterilized by immersion in 20% household bleach (50 g of active chlorine per liter) for 5 min, then thoroughly rinsed in deionized water and incubated at 25°C in the dark at saturation humidity until radicles were 2–5 mm long. Thereafter, individual sunflower seedlings were transplanted into pots with 250 g of a soil mixture SSP (sand:silt:peat moss 2:1:1, V). Pots were kept in a glasshouse at 12–22°C without additional lighting (15/9 h light/dark regime) for 5 weeks. Plants were watered as needed and, when they were 2 weeks old and until the end of the experiment 3 weeks later, they were fertilized once a week with 15 ml/pot of a nutrient solution with N:P:K (7:5:6).

### Inoculation with *O. cumana*, growth conditions and disease development

Sunflower plants were inoculated with population LPA13 of *O. cumana* (race F) (García-Carneros et al., 2014) following the methodology by Molinero-Ruiz et al. (2014). Eight sunflower seedlings (replications) of the inbred line NR5, which is the differential for the race F (Molinero-Ruiz et al., 2015), were individually transferred into pots with 250 g of SSP uniformly infested with 10 mg of parasite seeds. Eight non inoculated plants were established as controls. Plants were grown in the glasshouse under the same conditions described above for 5 weeks.

Sunflower plants were watched for development of wilting symptoms caused by the infection by *O. cumana*, but no visible differences were detected in inoculated plants as compared to the controls. At the end of the experiment, each plant was removed from the soil and its root system was washed and air dried at room temperature. Then, roots were weighed and the number and weight of *O. cumana* tubercles attached to the roots were subsequently recorded using a stereoscope and a precision balance, respectively.

### Blue-green fluorescence imaging

Blue-green fluorescence images were acquired sequentially with an Open FluorCamFC 800-O using UV (355 nm) excitation light. Images for F440 and F520 as well as images corresponding to the F440/F520 ratio were analyzed with Fluorcam7 software (Photon Systems Instruments, Brno, Czech Republic) according to Pérez-Bueno et al. (2015). Nine images were captured during 18 s in order to obtain each averaged fluorescence image. Image size was 640 × 480 pixels with a resolution of 96 pixels per inch. Measurements were taken on attached and unshaded leaves and always at the same time of day. Two replicates (one from each blade) were considered in each leaf pair (LP). Numerical data from a representative area in each replicate were analyzed.

The time span of BGF in healthy sunflower plants was determined by capturing images of the first four LPs each, every 3–4 days from 2 to 5-weeks-old. For each measurement date, LPs were independently analyzed, and between 6 and 8 replicates were considered for each of them.

Concerning the effect of *O. cumana* on the BGF emission of leaves of infected sunflower, measurements of F440 and F520, as well as those of F440/F520, F440/F680, and F440/F740, were acquired in all leaves of control and inoculated plants twice a week since they were 1 cm long until their complete expansion. Measurements were made during the V1–V4 vegetative stages of the plants (Schneiter and Miller, 1981) and, at each point in time, leaves of the same developmental stage were compared.

### Spectrophotometrical determination of pigments concentration in leaves

Total chlorophyll [Chl (*a*+*b*)] and carotenoids (xanthophylls and carotenes) [Car (x+c)] contents of parasitized sunflower were determined by spectrophotometry, and compared to those of the controls. Extractions were carried out on the second, third and fourth LP of the plants 5 weeks after inoculation (wai). From each LP blade, a 1 cm disk was punched, weighted and placed in vials containing liquid nitrogen. Disks were ground and photosynthetic pigments were extracted by adding 4 ml of 80% acetone (v/v). Absorbance at 470, 647, and 663 nm was measured from the resulting extracts with a Shimadzu UV-1800 spectrophotometer (Shimadzu Corporation, Tokyo, Japan). Concentrations of Chl *a*, Chl *b* and Car (x+c) were calculated according to Lichtenthaler and Buschmann (2001) and expressed as μg/g fresh leaf weight:

$$\begin{array}{lll} Chl_{a}\left(\mu g/ml\right) & = & 12.25A_{663.2}\;-\;2.79A_{646.8}\\ Chl_{b}\left(\mu g/ml\right) & = & 21.50A_{646.8}\;-\;5.10A_{663.2}\\ Car_{\left(x+c\right)}\left(\mu g/ml\right) & = & \left(1000A_{470}\;-\;1.82C_{a}\;-\;85.02C_{b}\right)/198 \end{array}$$

The Chl (*a*+*b)*/Car (*x*+*c*) ratio was calculated according to Konanz et al. (2014).

### Thermal imaging

Infrared images of leaves of inoculated and control sunflowers were taken using a FLIR A305sc camera (FLIR Systems, Wilsonville, Oregon, USA) that operates in the 7.5–13.5 μm wavelength range and has a thermal sensitivity < 0.05°C at +30°C and accuracy of ±2°C. The thermal camera was vertically positioned at approximately 0.3 m from the canopy and produced images of 320 × 240 pixels, with a viewing field of 45°. Digital video data were analyzed by the Research & Development software by FLIR.

Images were taken twice a week, from 2 and until 5 wai. At each time point, all fully developed leaves in control and in inoculated plants were measured. Measurements were performed on attached and unshaded leaves at the same time of day. Two hours before each measurement time, inoculated and control plants were moved from the glasshouse to a growth chamber in order to avoid glasshouse thermal fluctuations.

Digital color (RGB) images (2,048 × 1,536 pixels) were obtained simultaneous to the thermal images. One representative area in the midsection of each leaf was selected for the analysis after comparison of thermal and RGB images.

### Data processing and statistics

Data of the spatial distribution and time span of BGF on healthy sunflower were analyzed as means and their corresponding standard errors. When *O. cumana* was inoculated to sunflower, BGF emission, pigments concentration, leaf temperature and broomrape incidence [BI, transformed according to sqrt (BI + 0.5)] were statistically analyzed. The experiment was set up as a completely randomized design and was conducted twice. Similarity between repetitions was tested by analysis of variance (ANOVA) with repetitions as blocks. Since no significant differences were found, the data were pooled. When, after ANOVA, differences of any of the considered variables between inoculated and control plants were significant, mean values were compared by Fisher's protected Least Significant Difference tests (*P* = 0.05). STATISTIX 8.0 software (Analytical software, Tallahassee, FL, USA) was used for all the analyses.

## Results

### Blue-green fluorescence emission in healthy sunflower plants

**Figure 1:**
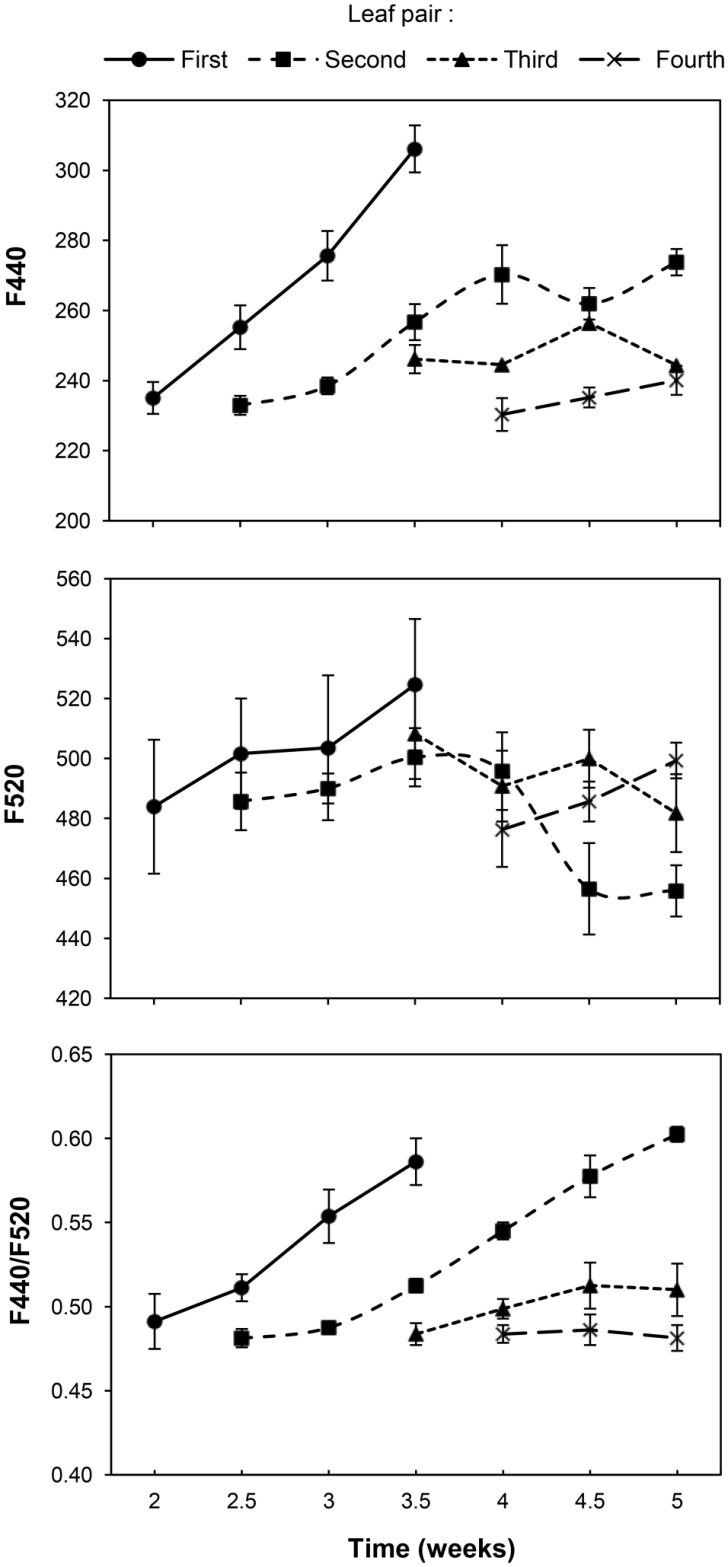
**Progress of blue-green fluorescence emission of the first, second, third and fourth pair of true leaves of a healthy sunflower plant**. Error bars represent the standard error of the mean of 8 replications. Time is expressed as weeks after inoculation by transplant.

A clear increase in the intensity of F440 and F440/F520 was observed in the first LP during its expansion and development. A similar trend was also observed in the case of the second LP in both parameters from the third week onwards. Values of F440 and F440/F520 of the two upper LPs evolved similarly to those of the lower LPs along the time, although smaller increases were observed in the third and fourth LP throughout the measurement period. By contrast, and with the exception of a slight increase in the F520 signal in the first LP, that of the rest of leaves remained fairly constant throughout the experiment, and even a decrease was detected in the second LP in the last week (Figure 1).

### Effect of *O. cumana* on BGF emission of sunflower

**Figure 2:**
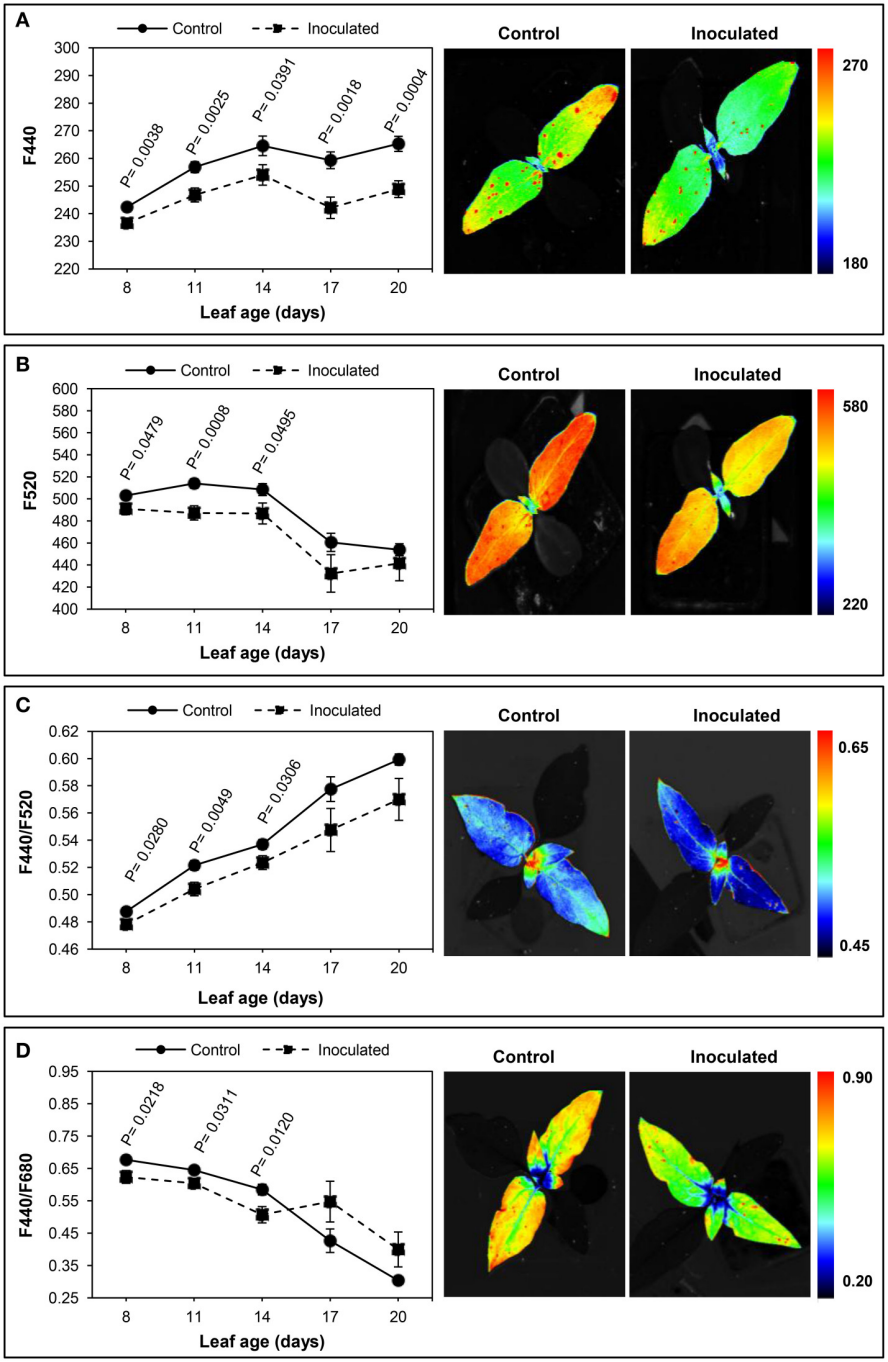
**Time-course of multicolour fluorescence emission of *Orobanche cumana-*inoculated sunflower plants and non-inoculated (control) plants throughout leaf development**. Mean measurements of F440 **(A)**, F520 **(B)**, F440/F520 **(C)**, and F440/F680 **(D)** of leaves and representative fluorescence images of 8 day-old leaves. Vertical bars represent the standard error of means of 14–48 replications. Analyses of variance at *P* < 0.05 between inoculated and control plants were conducted and values of significance are shown.

The effect of the infection of sunflower by *O. cumana* on the BGF emission of leaves was examined by comparison with that of leaves from the control plants at five moments during the 3 week period, and is presented in Figure 2.

A significantly lower fluorescence at 440 nm was consistently detected in parasitized plants throughout the experiment. Initially, leaves showed lower values of F440 than those at the end of the experiment, in both inoculated and control sunflowers (Figure 2A). Similarly, the F520 values in young leaves were significantly decreased by the parasite attack. As with F440, leaves of inoculated plants had lower F520, although F520 of older leaves did not significantly differ from that of the controls (Figure 2B).

Concerning the F440/F520 ratio, significant decreases were obtained in up to 14-day-old leaves of infected plants as compared to leaves from healthy plants. A progressive increase in F440/F520 ratio was evident in leaves of inoculated and control sunflowers throughout the experiment, although no significant differences were found between treatments in the two last measurements (Figure 2C).

On the other hand, the effect of the inoculation by *O. cumana* was also observed in the F440/F680 emission, being this fluorescence variable significantly low in 8 to 14-day-old leaves of parasitized sunflowers. Higher F440/F680 ratios were obtained in older leaves of inoculated plants, although differences with those in leaves of the control plants were not significant (Figure 2D). Similar results were obtained in the case of the F440/F740 ratio, young leaves of inoculated plants presenting significantly lower values than those of the control plants. When leaves were older than 14 days, the F440/F740 ratio was higher in the inoculated sunflowers, but no significant differences were found (data not shown).

### Carotenoids and total chlorophyll contents in sunflower leaves upon infection by *O. cumana*

The effect of the inoculation by *O. cumana* on chlorophyll and carotenoids contents at the end of the experiment is presented in Table 1. As previously observed by our research group (Ortiz-Bustos et al., 2016a), significant differences of chlorophyll content occurred in sunflower plants upon inoculation with *O. cumana*. Conversely, on the second and third LPs, no significant differences in either the carotenoids content or the chlorophyll/carotenoids ratio were detected between inoculated and control plants. Significantly lower values of both variables were obtained in the fourth LP of inoculated plants (203.20 μg/g and 5.07 for total carotenoids and for the chlorophyll/carotenoids ratio, respectively) compared with those in the same LP of healthy plants (274.30 μg/g and 5.80 for total carotenoids and for the chlorophyll/carotenoids ratio, respectively) (Table 1).

**Table 1:** **Measurements of pigments concentration (chlorophyll content, carotenoids content and chlorophyll/carotenoids ratio) for the second, third, and fourth leaf pair of sunflower plants inoculated with *Orobanche cumana* (I) and control plants (C) at 5 weeks after inoculation**.

**Pigments Concentration^a^**	**Treatment**	**Leaf pair (LP)**		
		**2nd**	**3rd**	**4th**
Chl (*a*+*b*)/Fw (μg/g fresh weight)	C	1359.50 ± 56.58^b^	1443.90 ± 51.90	1442.00 ± 101.04
	I	1548.60 ± 63.53	1515.90 ± 94.95	1028.40 ± 67.51
	*P*^c^	0.0462	0.5184	0.0052
Car (x+c)/Fw (μ g/g fresh weight)	C	239.96 ± 8.24	244.74 ± 15.84	274.30 ± 19.40
	I	263.57 ± 16.01	257.71 ± 22.38	203.20 ± 12.13
	*P*	0.2141	0.6434	0.0091
Chl (*a*+*b*)/Car (x+c)	C	5.92 ± 0.11	6.00 ± 0.17	5.80 ± 0.11
	I	5.93 ± 0.16	5.75 ± 0.22	5.07 ± 0.17

a Chlorophyll and carotenoids (xanthophylls and carotenes) content is expressed as content of chlorophyll a and b [Chl (a+b)], and [Car (x+c)] respectively, and chlorophyll/carotenoids ratio is expressed as Chl (a+b)/Car (x+c).

b Mean ± standard error (SE), n = 7–8.

c *Level of significance of differences in the variables between control (C) and inoculated (I) plants obtained after analyses of variance according to a completely randomized statistical design*.

### Effect of *O. cumana* on the temperature of sunflower leaf

**Figure 3:**
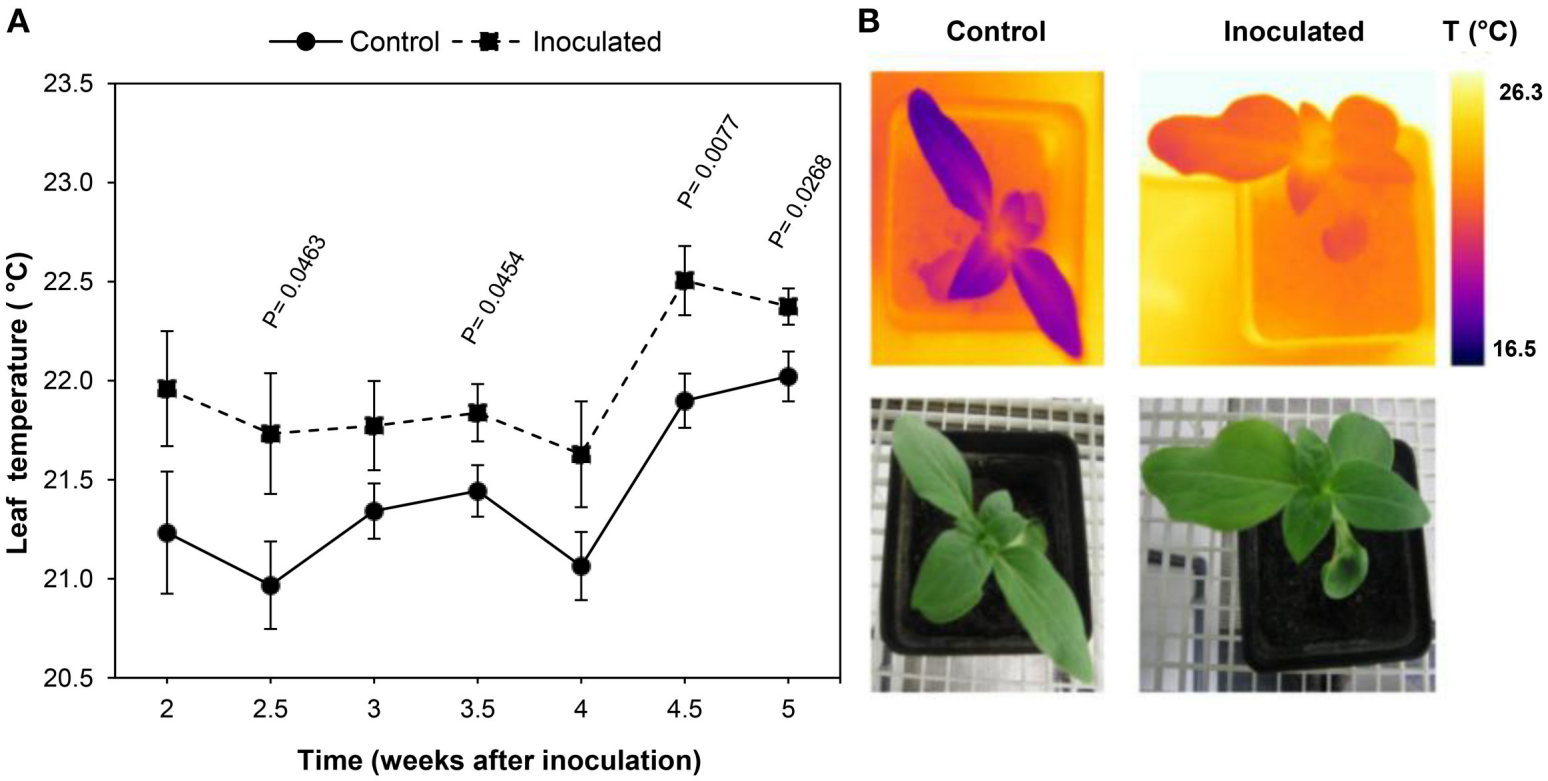
**Temperature progress of leaves of *Orobanche cumana-*inoculated sunflower plants and non-inoculated (control) plants. (A)** Mean measurements of leaf temperature of both treatments. Vertical bars represent the standard error of means of 16–45 replications. Time is expressed as weeks after inoculation. **(B)** RGB and thermal images of the first two leaf pairs of inoculated and control plants 2.5 weeks after inoculation.

Figure 3 shows the progress over time of the average leaf temperature of the inoculated and control plants.

Leaves of inoculated sunflowers were warmer than those of the controls at all the measurement dates. Significant temperature increases were observed 2.5 wai and persisted until the end of the experiment (5 wai) with the exception of measurements at 3 and 4 wai. Differences of leaf temperature ranged from 0.4 to 0.8°C at 5 and 2.5 wai respectively (Figure 3).

Leaf temperature showed an irregular time course irrespective whether the plants were inoculated or not, although a similar trend was observed in both situations. Thus, leaf temperature remained fairly constant during the two first weeks until 4 wai, when a clear increase was observed in the inoculated and also in the control leaves of sunflower (0.8 and 0.9°C respectively).

### Assessment of the infection of *O. cumana* in sunflower

Parasitized sunflowers reached the same growth stage than the controls. No symptoms (i.e., wilting) due to *O. cumana* were observed in aboveground parts of the plants throughout the experiment. Nonetheless, the infection was confirmed by tubercle formation in roots of inoculated sunflowers at the end of the experiment.

Although no significant differences in root weight were observed between inoculated and control plants, up to 0.29 g and 17 tubercles per plant were recorded in inoculated sunflowers. Tubercles were absent in the controls (Table 2).

**Table 2:** **Effect of *Orobanche cumana* infection on roots of sunflower and on the development of parasite tubercles 5 weeks after inoculation**.

**Treatment**	**Root fresh weight (g)**	**Tubercle fresh weight (g/plant)**	**No. tubercles per sunflower plant**
C	1.511 ± 0.265^a^	0 ± 0.000	0 ± 0.000
I	1.163 ± 0.176	0.2932 ± 0.115	17.38 ± 4.132
*P*^b^	0.2926		

a Mean ± standard error, n = 8.

b *Level of significance of differences of root fresh weight between control (C) and inoculated (I) plants obtained after analysis of variance according to a completely randomized statistical design*.

## Discussion

Our results showed that the BGF emission of individual LPs of healthy sunflower increases in time. Besides, the BGF emission depends on leaf development: the same pattern of the blue-green signal emitted by a LP (i.e., the first LP) until its complete expansion was presented, 1 week later, by the following LP (i.e., the second LP). An increase of BGF of leaves throughout their elongation was observed in wheat by Meyer et al. (2003), who proposed BGF as a signature of leaf aging. Indeed, aging of leaf implies transition from source to sink tissue by diversion of carbon from primary to secondary metabolism. On the other hand, different BGF emissions in artichoke leaves with age were also observed by Morales et al. (2005), although old leaves emitted lower BGF than young ones. Interestingly, although in most plant species ferulic acid is the major blue-green fluorophore (Morales et al., 1996), in the case of sunflower there is no ferulic acid bound to cell walls. Small amounts of caffeic acid and trichome secretions (e.g., sesquiterpenes lactones and coumarins) have been described as blue-green fluorophores in sunflower (Spring and Schilling, 1989; Olson and Roseland, 1991; Morales et al., 1996; Tourvieille de Labrouhe et al., 1997; Lichtenthaler and Schweiger, 1998; Rowe et al., 2012). These compounds could be responsible for BGF emission in our experiments. In fact, the coumarins content in sunflowers grown under optimal non-limiting conditions tends to be very low, thus their presence can be considered as a good marker of a stress event (Prats-Perez et al., 2000).

Apart from monitoring plant physiological status in response to growth, BGF has been successfully used to detect nutrient deficiencies (McMurtrey et al., 1996; Cadet and Samson, 2011), water and temperature stress (Lang et al., 1996; Hura et al., 2007), pathogen attack (Granum et al., 2015) and a simultaneous combination of stress events (Bürling et al., 2011). Preliminary studies by our group proved the potential of BGF to discriminate *O. cumana* infection in sunflower plants at early stages (3 wai) (Pérez-Bueno et al., 2014). The present findings allow us to distinguish infected from non-infected sunflowers 1 week earlier by means of decreases in F440 and F520 emissions, as well as by lower values of the F440/F520, F440/F680, and F440/F740 ratios. Spectral features of BGF and their intensity ratios have been investigated not only for their diagnostic value, but also for understanding the physiological changes that take place during stress development (Buschmann and Lichtenthaler, 1998). Our BGF imaging results suggest that *O. cumana* alters the secondary metabolism of sunflower, e.g., accumulation of caffeic acid and coumarins. Furthermore, the spectrophotometric pigment quantification provided evidence of decreased contents of carotenoids in leaves of infected plants as compared to those of the controls in agreement with the results by Shen et al. (2013) on *Mikania micrantha* infected by *Cuscuta campestris*. Many secondary metabolites are not only major contributors to specific odors, tastes, and colors of plants, but they also play a key role in defense against herbivores and pathogens (Berger et al., 2007; Wink, 2010; Ouzounis et al., 2015). Coumarins are excreted by roots of sunflowers with resistance to the parasite (Serghini et al., 2001), as well as other toxic (Zélicourt et al., 2007) and phenolic (Echevarría-Zomeño et al., 2006) compounds, all of them having a defensive role against *O. cumana*.

Since the F440/F520 ratio is affected by chlorophyll content (Stober and Lichtenthaler, 1992; Morales et al., 1994), decreases upon infection by *O. cumana* suggest that a lower content of chlorophyll is present in leaves. Recent findings by our research group also suggested that, when sunflowers are infected by *O. cumana*, the chlorophyll content in young leaves is decreased (Ortiz-Bustos et al., 2016a). Reductions in chlorophyll content are also induced by *O*. *foetida* attack on chickpea (*Cicer arietinum* L.) (Nefzi et al., 2016) and by *C. australis* infection on *M. micrantha* (Le et al., 2015). Our results also support the value of the fluorescence ratios F440/F680 and F440/F740 as very early stress indicators (Lichtenthaler and Miehé, 1997; Buschmann and Lichtenthaler, 1998; Buschmann et al., 2000). Low values of these ratios during early stages of the infection of sunflower may not only be due to decreased F440 and F520 emission in leaves, but also to higher F680 and F740 signals (Ortiz-Bustos et al., 2016a). Beyond that, our findings show that BGF and its intensity ratios with chlorophyll bands could be used, in addition to directly detecting *O. cumana* in sunflower, as an indirect approach to the alteration of plant photosynthesis and to the impairment of the secondary metabolism as a result of parasite infection.

The applicability of thermal infrared imaging in determining plant temperature as an early response to biotic or abiotic stresses is widely documented and a topic of hectic research activity (Nilsson, 1995; Chaerle and Van Der Straeten, 2001; Raza et al., 2014; Baranowski et al., 2015; Grant et al., 2016; Mahlein, 2016; Mangus et al., 2016). In this work we observed increases in leaf temperature of parasite infected sunflower from 2 to 5 wai. Recently, increases in canopy temperature allowed the detection of late wilt disease caused by the soil borne fungus *Harpophora maydis* up to 17 days earlier than symptoms development in maize (Ortiz-Bustos et al., 2016b). Also, significant increases in crown temperature have allowed the differentiation of olive trees infected by *Verticillium dahliae* in the field (Calderón et al., 2013). Leaf thermal increases are closely related to reduced transpiration rates due to either activation of stomatal closure or inhibition of stomatal opening. Previous studies revealed that *Orobanche ramosa* and *C. campestris* parasitism reduced stomatal conductance and transpiration rate and, consequently, slowed down host photosynthesis and host growth (Mauromicale et al., 2008; Chen et al., 2011). The effects of parasite-induced stomatal closure and transpiration reduction on the decreased development of sunflower (Alcántara et al., 2006) should be investigated in the future. To the best of our knowledge, this work constitutes the first approach to the diagnosis of parasite infection in crops by means of thermal imaging and could be further implemented in field conditions.

Although the damage done by broomrape species to crops is directly attributed to parasitic sink activity (Barker et al., 1996; Manschadi et al., 1996; Hibberd et al., 1998; Draie et al., 2011; Péron et al., 2012), our results have evidenced that, in the *O. cumana*—sunflower interaction, the damage might extend beyond assimilate diversion. Many parasitic angiosperms display a pathogenic nature promoting disease in the crop mainly through negative effects on the photosynthesis, physiology and hormonal balance of the host (Stewart and Press, 1990; Watling and Press, 2001; Mauromicale et al., 2008). The present work provides valuable and essential clues toward the understanding of the processes by which *O. cumana* seems not only to cause changes in sunflower secondary metabolism but also to alter its photosynthetic capacity and unbalance its carbohydrate metabolism. Nevertheless, additional research will be required to clarify how both physiological processes are affected.

## Conclusion

The outstanding significance of our BGF imaging and thermography results, from a diagnosis point of view, is that the establishment of a soil borne pathogen (*O. cumana*) in a below-ground organ (root) of the plant can be detected prior to the development of visual symptoms in far distant and above-ground organs (leaves). Diagnostic fluorescence and thermal signals are related to host physiology alterations upon infection, and continuous measurements for long periods of time are possible. Therefore, these techniques enable not only the detection of stress onset by *O. cumana*, but also the monitoring of its development in sunflower over time, providing an additional tool for basic research about holoparasite-host plant interactions. Finally, and as a useful outcome of this work, a fast phenotyping of sunflower lines could be achieved by means of the implementation of BGF imaging and thermography in breeding programmes for resistance to *O. cumana*.

## Author contributions

LM, MP, and MB conceived and designed the experiments. CO, LM, and MP conducted experiments. CO and LM analyzed data and interpreted the results. CO mounted images. LM and MB contributed materials, equipment and analysis tools. CO and LM wrote the manuscript and all the authors reviewed it and approved the final version.

### Conflict of interest statement

The authors declare that the research was conducted in the absence of any commercial or financial relationships that could be construed as a potential conflict of interest.
